# How big is the elephant in the room? Estimated and actual IT costs in an online behaviour change trial

**DOI:** 10.1186/1756-0500-3-172

**Published:** 2010-06-18

**Authors:** Jim McCambridge, Orla O'Donnell, Christine Godfrey, Zarnie Khadjesari, Stuart Linke, Elizabeth Murray, Paul Wallace

**Affiliations:** 1 Centre for Research on Drugs & Health Behaviour, London School of Hygiene & Tropical Medicine, Keppel Street, London WC1E 7HT, UK; 2 E-health Unit, Research Department of Primary Care & Population Health, UCL, UK; 3 Department of Health Sciences and HYMS, University of York, UK

## Abstract

**Background:**

The practical and methodological challenges inherent in online behaviour change studies are both novel and complex. We relate our experiences of estimating and managing information technology (IT) research and intervention costs in an ongoing internet trial in the hope that others will find this information useful.

**Findings:**

Actual IT costs were approximately twice those that had been originally estimated by external contractors. These original estimates for IT costs allowed little scope for the identification of new needs, which was intrinsic to the iterative nature of the research enterprise.

**Conclusions:**

Making greater provision for the uncertain nature of these costs in future studies is a key practical lesson for the planning of future online behaviour change studies.

## Background

Estimating research and intervention costs in the planning of any large study is a considerable practical challenge facing researchers. Overestimation may threaten the granting of research funding and underestimation can threaten the viability, quality and completion of the study. For most trials costs can be divided between those required to develop the intervention materials (both active and control) and the research evaluation. There is a particular need to take account of both the practical and methodological challenges inherent in online studies as the internet is increasingly used to both deliver healthcare and public health interventions, and to undertake research[1]. Here the uncertainties unavoidably encountered in any relatively new field of study are acute, and this raises additional technical and financial issues. We relate our experiences of estimating and managing information technology (IT) research and intervention costs in an ongoing internet trial in the hope that others will find this information useful.

## The Down Your Drink Trial as a case study

The Down Your Drink randomised controlled trial[2] was funded by the U.K. Medical Research Council on behalf of the National Prevention Research Initiative (see acknowledgements for further details). This ongoing study compares the effectiveness of an enhanced website in helping people concerned about their drinking to achieve behaviour change[3] in comparison with a standard alcohol website. The enhanced website was developed from an existing website which had been in operation for some years,[4] with demonstrated potential for effectiveness[5]. Thus IT costs presented here relate to the further development of an existing website for the "intervention" components of the trial and the new additional requirements related to the research evaluation. In practical terms, it was these additional costs that need to be estimated at the research proposal stage. For the research and for its economic evaluation component it is also necessary to be able to separate out research based costs (not included in the economic evaluation) from the intervention costs (which do need to be estimated and included). This will identify the actual costs of developing and maintaining the website to be incorporated into cost-effectiveness analyses.

Our chosen modus operandi involved a technical consultant liaising between the research team and the programmers who were based in a small private sector IT company. A competitive tendering process had occurred for the original establishment of the website some years previously and it would have been difficult for technical reasons to involve new personnel. As it was essential to have current expertise on web design and the user interface, we opted to work with the private sector rather than with an academic IT department. For this case study the use of a private company had the advantage that resources used in the trial were more specifically monitored than may have been the case in a large academic department. Communications between the various parties were effective, though constrained in ways to be described. We examined the literature for guidance on IT costs and had informal discussions with other e-health researchers, but at that time (during 2006) there was little collective experience of on-line trials. Some of the costs we present are specific to our model and our circumstances, though the lessons drawn from this experience are more clearly generalisable.

In a total research grant awarded of approximately £350,000* over three and a half years, we were awarded £21,400 for website development and maintenance costs, exactly as requested (N.B. all totals include Value Added Tax [VAT]; a general sales tax, which at the time of the study was 17.5%). We received £15,400 for website development and maintenance and £6,000 for the technical consultant who also fulfilled a project management function. These costings were based on estimates we received from both external contractors in the course of the research grant application, and before we started the work on redeveloping the existing website. Our funding award involved a pilot study being undertaken online 3 months after the initiation of the project, leading into a main trial after approximately one year.

## IT Resource Use in Practice

After the project was initiated, a work plan was put together to provide a more detailed specification of how the awarded funds were to be initially spent -- see Table 1. The IT costs in Table 1 comprise work which was needed to begin the pilot study and for it to provide a thorough piloting of our trial procedures. We were aware, at this point, that there would be additional work to be done, though unaware of how much. An important issue that arose early was the need to separate the research and intervention and control websites as we gave detailed attention to the nature of the engagement of the research participant and the need to control a range of potential sources of bias. The possibility of performance bias induced by inadvertent compromise of equivalence between intervention and control groups required us to carefully consider exactly how the research processes engaged participants in each group. We used hypothetical participant "walk through" scenarios that allowed us to think through the likely unintended implications for the participant of implementing our proposed research design. We were particularly concerned about the potential for information bias in this online context, a subject on which there is a limited evidence base[6]. To provide additional assurance about the validity of self-reported data, and to prevent any such problems being differential between randomised groups, we sought to entirely separate website access for research data collection purposes from the interventions for the participant. This involved presenting the research and intervention websites wholly differently to each other. Whilst advance planning of the research at the proposal stage had taken some account of these issues, there had been no real opportunity to fully consider the issues involved.

**Table 1:** Initial detailed work and costing plan

	*Total (£)*
*Trial Website:*	
Implementation of the consent process and the randomization and group assignment system	470
Implementation of the primary and secondary outcome questionnaires, including the code	1,410
Basic e-mail reminders system and follow-up questionnaires system	1,645
*Control Website:*	
Implementation of the control site (estimated about 15-20 pages of information)	1,645
Intervention Website:	
Implementation of the three modules, based on restructuring some old content and adding more new content provided by research team	5,875
SUB TOTAL:	11,045
Technical consultancy & project management	7,050
AGREED SPEND AT OUTSET:	18,095
VAT @ 17.5%	3,167
**TOTAL**	21,262

These tasks were duly implemented. In so doing, it quickly became apparent that additional work would be required. This stemmed from the fundamentally iterative nature of the enterprise. Once particular tasks were completed, only then could it be established whether additional work was or was not needed. For example, testing new material with users was judged essential to the successful conduct of the study. It is difficult to know in advance, however, precisely what need for further work will emerge from this feedback and thus what the costs will be.

It should be emphasised that we were not striving to include the latest graphics and other technical innovations. All along we were mindful of the need to provide easy access to those with slow download speeds, and to make the entire process of trial participation and engagement with the interventions as straightforward as possible. Formative evaluations of progress made led in a series of further iterations to the identification of new needs for further work to be done. Information on these additional costs is presented in detail in Table 2. The majority of these additional costs involved refinements of the intervention website, almost entirely in response to feedback from users.

**Table 2:** Additional work required

	*Total (£)*
*Trial Website:*	
System to provide password protected downloadable data for the research team	1,410
Implementation of a participant withdrawal system	1,175
New randomization system	353
Incentives sub-study (incidental)	822
Changes to registration system following pilot study	235
Maintenance fee for the main trial	1,410
One Year follow-ups - implementation of the new incentives system	1,880
*Control Website:*	
There was no overspend on the development of the Control Website	
*Intervention Website:*	
Graphics	764
Modules development and content management system	4,465
Copyright fees for use of images	1,000
Additional features to enhance the attractiveness and functioning of the website	7,344
Set up standalone outcome measure site at http://tot-al.downyourdrink.org.uk (incidental)	176
*Consultancy costs:*	
Additional consultancy costs	1,410
SUB-TOTAL:	22,444
VAT @ 17.5%	3,928
**TOTAL**	26,372

Some developments were also initiated or required by other parties. For example, one participant who wished to withdraw from the study contacted the ethical committee because they no longer wished to be bothered by automated e-mail messages. The ethical committee required that this be done, and we were very happy to do it, though this did involve unforeseen programming costs. We sought and obtained additional funding support for the newly identified extra work (see acknowledgements). If we had not been able to obtain additional funding, the conduct of the study would have been jeopardised.

There are two other types of research costs described in Table 2. Firstly, there were costs for the main trial that emanated from the pilot study, in which we altered our research decisions on the basis of accumulating research data. Secondly, other costs were attributable to incidental studies. These were sub-studies which were undertaken to optimise the research decision-making for the main trial. For example, we conducted a number of experimental studies examining various methods to minimise attrition, a central problem in internet longitudinal research[7]. In both cases, the needs of the research study itself required unforeseen expenditures if it was to be done as well as possible.

Towards the end of the study, further work was identified to fulfil our initial commitment to make the website freely available after the end of the trial. This work was thus neither specifically an intervention nor a research cost and totalled £4,140. These were added to the total programming costs of £10,986 for research purposes, £19,448 on the development of the intervention website, £1,645 on the control website, and £8,460 on consultancy costs to give a final total for IT costs of £44, 679. Research-specific IT costs were much lower than were intervention IT costs, and costs for our Technical Consultant (which embraced both research and intervention activities) were far from trivial. Research staff costs have not been included here.

## Conclusions

In total the actual costs of the IT work were more than twice what had originally been estimated (i.e. compared to £21,400). It was almost as if the elephant in the room was twice as big as we thought it would be, notwithstanding our efforts to obtain well informed estimates. With the benefit of hindsight our original estimates allowed little scope for the identification of new IT needs, beyond the need for maintenance costs, and thus were grossly inadequate. Perhaps we could have given more scrutiny to the estimates we received from contractors. Even so, the fundamental problem that we faced was that many of the additional costs could not have been specifically anticipated in advance as the need for them only became apparent during the course of the methodological and intervention design work that was the substance of the research itself. And we were reliant on the estimates provided by external expert IT contractors, precisely because they had expertise which we did not.

Current research funding systems rely on academic departments taking the risk for any underestimation of resources, while most research funding bodies have mechanisms to claw back those resources not used. This risk is managed by most universities through some pooling of risks across different projects and different funding streams to support research activities. However, these systems do not usually extend to flexibility in external contracts for specialist services such as IT support.

There is a limited pool of knowledge about how to do studies of this type effectively and efficiently and we certainly do not suggest that our particular model, involving an external technical consultant, should be taken up more widely. Particularly noteworthy for internet intervention developmental work is the fact that we deliberately eschewed advanced interactive features and videos on grounds of cost and user engagement. We were also aware of the need to limit not just the direct IT costs but also the hidden costs of research project staff time devoted to IT issues. Although these are difficult to separate from wider research costs, they may well exceed the IT specific costs and it was our experience that such staff time was extended by the inevitable communication problems encountered in bridging the worlds of academic research and the internet in addressing ongoing issues. Making greater provision for the essentially unknowable nature of these costs in future studies is thus a key practical lesson for the planning of future internet behaviour change studies.

*The value of financial costs may be approximately converted to U.S. dollars or Euros by multiplying by 1.5 or 1.2 respectively, though exchange rates obviously do vary.

## Competing interests

The authors declare that they have no competing interests.

## Authors' contributions

JM wrote the first draft of the paper and led the writing process. OO gathered the costs data. OO, CG, ZK, SL, EM and PW provided input into revisions. All authors are involved in the parent trial from which this short report is derived and read and approved the final manuscript.
